# Postoperative Cognitive Dysfunction and Alzheimer’s Disease: A Transcriptome-Based Comparison of Animal Models

**DOI:** 10.3389/fnagi.2022.900350

**Published:** 2022-06-28

**Authors:** Yi-Wei Wang, Liang Wang, Sheng-Jie Yuan, Yuan Zhang, Xin Zhang, Le-Ting Zhou

**Affiliations:** ^1^Department of Anesthesiology, The Affiliated Wuxi People’s Hospital of Nanjing Medical University, Wuxi, China; ^2^Department of Internal Medicine, The Affiliated Wuxi People’s Hospital of Nanjing Medical University, Wuxi, China; ^3^Department of Anesthesiology, Center for Translational Pain Medicine, Duke University School of Medicine, Durham, NC, United States

**Keywords:** postoperative cognitive dysfunction, Alzheimer’s disease, bioinformatics, transcriptomics, high throughput data

## Abstract

**Background:**

Postoperative cognitive dysfunction (POCD) is a common complication characterized by a significant cognitive decline. Increasing evidence suggests an association between the pathogenesis of POCD and Alzheimer’s disease (AD). However, a comprehensive understanding of their relationships is still lacking.

**Methods:**

First, related databases were obtained from GEO, ArrayExpress, CNGB, and DDBJ repositories. *De novo* analysis was performed on the raw data using a uniform bioinformatics workflow. Then, macro- and micro-level comparisons were conducted between the transcriptomic changes associated with AD and POCD. Lastly, POCD was induced in male C57BL/6j mice and the hippocampal expression levels of mRNAs of interest were verified by PCR and compared to those in AD congenic models.

**Results:**

There was a very weak correlation in the fold-changes in protein-coding transcripts between AD and POCD. Overall pathway-level comparison suggested that AD and POCD are two disease entities. Consistently, in the classical AD pathway, the mitochondrial complex and tubulin mRNAs were downregulated in both the POCD hippocampus and cortex. POCD and AD hippocampi might share the same pathways, such as tryptophan metabolism, but undergo different pathological changes in phagosome and transferrin endocytosis pathways. The core cluster in the hippocampal network was mainly enriched in mitosis-related pathways. The hippocampal expression levels of genes of interest detected by PCR showed good consistency with those generated by high throughput platforms.

**Conclusion:**

POCD and AD are associated with different transcriptomic changes despite their similar clinical manifestations. This study provides a valuable resource for identifying biomarkers and therapeutic targets for POCD.

## Introduction

With the dramatic change in life expectancy (Granger and Barnett, 2021), more surgeries are being performed in progressively older adults. In the United States, approximately 35% of all operations are being performed on adults older than 65 years (Evered et al., 2017). There are proportionately more complications in older surgical patients than in younger patients, postoperative cognitive dysfunction (POCD) in particular (Evered et al., 2018). POCD is a neurological complication characterized by impaired memory, deficits in information processing, and reduced attention, accompanied by changes in mood and personality. Furthermore, POCD can persist years after surgery and is probably irreversible in many cases with an increasing overall morbidity and mortality (Subramaniyan and Terrando, 2019).

Alzheimer’s disease (AD) is the most common neurodegenerative disease that leads to cognitive decline in the elderly and is estimated to affect 26.6 million people worldwide (Scheltens et al., 2021). As cognitive deterioration is the same key feature of POCD and AD, it is reasonable to speculate that these two diseases share certain biological processes. Some reports have demonstrated that surgery and anesthesia cause the accumulation of amyloid β (Aβ) proteins and promote aberrant tau phosphorylation ***in vivo and in vitro***, which are pivotal pathological changes in the development of AD (Bilotta et al., 2010; Arora et al., 2014; Evered et al., 2017; Marques and Lapa, 2018). However, clinical trials have failed to observe the significant deterioration of cognition in patients with AD after surgery or anesthesia. This suggests that there is a complex association between the pathogenesis of POCD and AD. In this study, we performed a systematic bioinformatics analysis and verification of high-throughput transcriptomic data from both POCD and AD animal models to compare the underlying molecular mechanisms of these two diseases. We hypothesized that significant differences could be revealed, which would provide clues to accelerate the study of novel diagnostic biomarkers and therapeutic targets for POCD.

## Materials and Methods

### Bioinformatics Workflow and Dataset Collection

**FIGURE 1:**
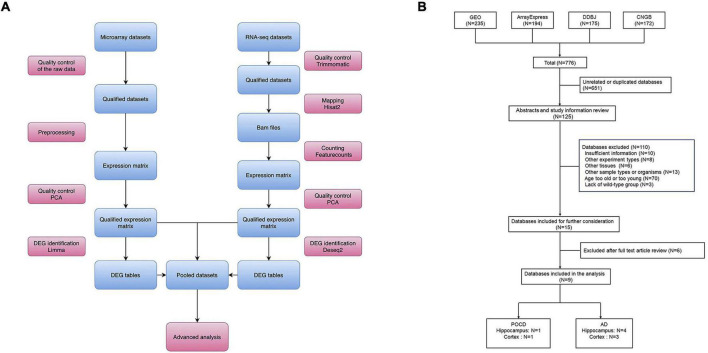
**(A)** Overall study design and bioinformatics analysis workflow. **(B)** Flow diagram of dataset search and selection.

The overall bioinformatics workflow is presented in Figure 1A. First, datasets were collected from the Gene Expression Omnibus (GEO^1^), ArrayExpress^2^, China National GeneBank DataBase (CNGB)^3^, and DDBJ^4^ in September 2021. The following search criteria were applied: [(POCD OR post-operative cognitive dysfunction OR PND OR perioperative neurocognitive disorders)] OR (AD OR Alzheimer’s disease). Transcriptomic datasets from the mouse hippocampus or cortex, generated using microarray or RNA-sequencing (RNA-seq) platforms, were analyzed. The summary and sample description of each dataset were carefully evaluated by two investigators before inclusion.

### Raw Data Processing

Raw data processing was performed using R (3.6.1) and Linux shell. In summary, the main steps of data preprocessing included quality control of the raw data, calculation of the expression matrices, and quality control of the expression matrices (Figure 1A). Qualified matrices were included in the analyses. The robust multichip average algorithm (affy package), the normexp and quantile method (limma package), and the neqc function (limma package) were applied to generate the expression matrices from the Affymetrix, Agilent, and Illumina raw microarrays, respectively. The HISAT2-featureCounts workflow was applied for mapping and counting the RNA-seq datasets with GRCm38 (Mus musculus) used as the reference genome. For platforms with no valid annotation file, Rsubread and GenomicRanges packages were applied for probe re-annotation. All non-protein-coding genes were removed using the BiomaRt package in R.

### Differentially Expressed Gene Identification and Correlation Analysis

The log_2_ fold-changes and *p*-values were calculated using the negative binomial distribution-based count model in the DEseq2 package for RNA-seq datasets, and the empirical Bayes model in the limma package for microarray datasets. The Benjamini–Hochberg method was used to adjust the p-values. Statistically significant differentially expressed genes (DEGs) were defined as those with the lowest deciles of adjusted *p*-values to avoid introducing bias from different platforms and sample sizes. Spearman’s correlation analysis was performed to calculate the correlation coefficients. The generalized additive model was applied to fit the results and to evaluate the similarities between all evaluated transcriptomes.

### Over-Representation Analysis

This was performed using clusterProfiler (version 3.12.0) and ggplot2 (version 3.3.2) packages in R. Specifically, Gene Ontology (GO) annotation^5^, Kyoto Encyclopedia of Genes and Genomes (KEGG) pathway^6^, and Reactome pathway^7^ queries were realized via the Annotation Hub, KEGG Pathway Module, and ReactomePA, respectively. A bubble plot was used for visualization. A hypergeometric test was used for enrichment analysis. Statistical significance was set at *p* < 0.05.

### Gene Set Enrichment Analysis

This was implemented using the clusterProfiler package in R with inputs of the ranked gene lists based on the log_2_ fold-change. The curated gene set from the Broad Institute’s Molecular Signature Database (MSigDB; C2:KEGG) was queried and transformed into mouse versions using the msigdbr package. Subsequently, 1,000 permutations were performed to generate significant p-values, which were defined as those <0.05 without multiple hypothesis testing. For the single-term analysis, the enrichment plot and pathview package in R were utilized to generate gene set enrichment analysis plots and topology-based pathway plots, respectively.

### Pathway Level Comparison Based on Gene Set Variation Analysis

This was performed in accordance with Ramsey (2018). Briefly, the minimum expression-level cutoff was initially calculated using the kernel density estimation for each dataset. Genes that were not expressed based on the minimum expression level cutoff were removed from the expression matrix. Gene set variation analysis was used to map the gene expression data to pathway-level matrices, and the KEGG database was used for pathway annotation. Finally, the gene set variation analysis-transformed data were merged and visualized using principal coordinate analysis and principal component analysis.

### Meta-Analysis

This was performed using the robust rank aggregation package in R, which implements a robust rank aggregation method to combine the results from different studies (Kolde et al., 2012). This method uses a probabilistic model, which makes it robust to outliers and noise. The final list was ranked according to significance scores. Significantly dysregulated genes were defined as those with the lowest deciles of significance scores.

### Constructing the Protein–Protein Interaction Network, Hub Gene Recognition, and Cluster Analysis

Protein–protein interaction (PPI) network (PIN) analysis was performed using the STRING database^8^. Edges with a combined score > 0.4 were included in the topological analysis. The average local clustering coefficient and PPI enrichment values were applied to measure the network connections. Cytoscape (version 3.9.0) was used to visualize the PIN and analyze the characteristics of each node. The maximal clique centrality algorithm in the CytoHubba plug-in was used to identify hub genes (Chin et al., 2014).

### Animals and Surgery

Male C57BL/6j (*n* = 10), congenic 5 × FAD (*n* = 5), 3 × TgAD (*n* = 5), and APP/PS1 (*n* = 5) mice were provided by the Jiangsu Animal Experimental for Medical and Pharmaceutical Research Center. All animals were housed under specific pathogen-free conditions until 12 months of age. After acclimation for at least 1 week, animals were used in experiments. All animal experiments in this study were approved by the Institutional Animal Care and Use Committee (Approval No.: 2003021) and the Laboratory Animal Ethics Committee of Nanjing Medical University.

Male C57BL/6j mice (12 months old) were randomly assigned into control and POCD groups. Exploratory laparotomy under isoflurane anesthesia (1.5% isoflurane mixed with oxygen at 2 L/min) was used to construct the POCD model (Qiu et al., 2020). At the end of the procedure and the following 3 days, 2.5% lidocaine cream was applied to the incision to alleviate surgery-associated pain. Our experiment conformed with the guidelines laid down by the NIH regarding the care and use of animals for experimental procedures. Methodological details are described in the Supplementary Materials.

### Behavioral Tests and Tissue Processing

The behavioral tests included the Morris water maze (MWM) and trace fear conditioning (TFC); methodological details are described in the Supplementary Materials. One hour after the tests, all mice were deeply anesthetized with isoflurane and euthanized by exsanguination. The brain was extracted to obtain the hippocampus; the tissues were then frozen in liquid nitrogen for RNA isolation and reverse transcription.

### Quantitative PCR

Total RNA from the hippocampus was extracted with TRIzol reagent (Invitrogen, Carlsbad, CA, United States), and reverse transcription was performed using the SuperScript III first strand synthesis system (Invitrogen, Carlsbad, CA, United States). Quantitative PCR (qPCR) amplification was performed using the STEP ONE Real Time PCR Detection System with SYBR Green master mix (Applied Biosystems, Foster City, CA, United States). Methodological details are described in the Supplementary Materials.

### Statistical Analysis

The data were first tested for normality (Shapiro–Wilk test) and homoscedasticity (Levene’s test). All data are presented as either the mean ± SEM or as the median and interquartile range. Repeated measures analysis of variance (ANOVA) was performed to compare the escape latency between the control and POCD groups. For other comparisons, either a *t*-test (for two groups) or one-way ANOVA (for more than two groups) was applied. The *post-hoc* tests for one-way ANOVA were performed either using the least significant difference (if the variance was equal) or Dunnett’s T3 (if the variance was not equal) tests. Statistical significance was set at a two-sided *p-*value < 0.05.

## Results

### Characteristics of Datasets

In total, 776 datasets were identified from the GEO, ArrayExpress, DDBJ, and CNGB genomics data repositories (Figure 1B). Among these, 125 were retained for further assessment after removing 651 duplicate datasets. After reviewing the abstract and full text, nine datasets comprising 126 samples (AD hippocampus, *n* = 4; AD cortex, *n* = 3; POCD hippocampus, *n* = 1; POCD cortex, *n* = 1) were included in the final analysis (Figure 1B). These datasets were generated by Affymetrix, Agilent, and Illumina microarrays, and Illumina RNA-seq platforms (Table 1).

**TABLE 1:** Dataset characteristics.

GEO accession	Organism	Tissue	Experiment type	Disease model	Sex	Age (months)	Extracted molecule
GSE95426	Mus musculus	Hippocampus	Expression profiling by array	POCD mice	Male	12–14	Total RNA
GSE174412	Mus musculus	Cortex	Expression profiling by high throughput sequencing	POCD mice	Male	18	Total RNA
GSE135999	Mus musculus	Hippocampus	Expression profiling by array	APP/PS1 AD mice	Male/Female	12	Total RNA
GSE135999	Mus musculus	Cortex	Expression profiling by array	APP/PS1 AD mice	Male/Female	12	Total RNA
GSE165111	Mus musculus	Hippocampus	Expression profiling by array	3xTg AD mice	Male/Female	15–20	Total RNA
GSE93678	Mus musculus	Hippocampus	Expression profiling by high throughput sequencing	APP/PS1 AD mice	Female	13	Total RNA
GSE168137	Mus musculus	Hippocampus	Expression profiling by high throughput sequencing	5xFAD AD mice	Male/Female	18	Total RNA
GSE168137	Mus musculus	Cortex	Expression profiling by high throughput sequencing	5xFAD AD mice	Male/Female	18	Total RNA
GSE60911	Mus musculus	Cortex	Expression profiling by array	3xTg AD mice	Female	20	Total RNA

Mice from the POCD group underwent tibial fracture surgery (GSE95426) or exploratory laparotomy (GSE174412), whereas AD mouse models included the 3 × Tg, 5 × FAD, and APP/PS1 models. All samples were collected from aged mice (12–20 months old). The resources of the datasets included GSE95426 (citation missing in GEO), GSE174412 (Wu et al., 2021), GSE135999 (Hou et al., 2021), GSE165111 (Kim et al., 2021), GSE93678 (Daugherty et al., 2017), GSE168137 (Forner et al., 2021), and GSE60911 (Sykora et al., 2015). All raw data were analyzed in accordance with the workflow described in the Methods section.

### Postoperative Cognitive Dysfunction vs. Alzheimer’s Disease: Correlation Analysis

**FIGURE 2:**
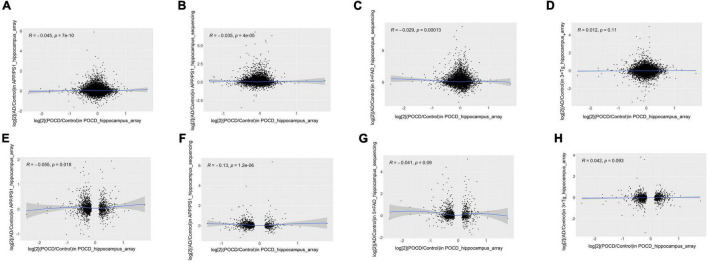
Correlation analysis of all **(A–D)** and significantly dysregulated **(E–H)** protein-coding gene expression fold-changes between POCD and different AD transgenic models in the hippocampus. **(A)** GSE95426 (POCD model microarray data) vs. GSE135999 (APP/PS1 model microarray data); **(B)** GSE95426 vs. GSE93678 (RNA-seq data from APP/PS1 model); **(C)** GSE95426 vs. GSE168137 (RNA-seq data from 5 × FAD model); **(D)** GSE95426 vs. GSE165111 (3 × Tg model microarray data). **(E)** GSE95426 (POCD model microarray data) vs. GSE135999 (APP/PS1 model microarray data); **(F)** GSE95426 vs. GSE93678 (APP/PS1 model RNA-seq data); **(G)** GSE95426 vs. GSE168137 (5 × FAD model RNA-seq data); **(H)** GSE95426 vs. GSE165111 (3 × Tg model microarray data).

Fold-change-based transcriptome-wide correlation analysis was performed to compare the overall similarity. First, the correlations of all protein-coding genes in the hippocampus were analyzed between POCD and the different AD models regardless of their statistical significance. A poor correlation was determined between POCD and all AD models (APP/PS1 microarray: Figure 2A, Spearman’s *R* = −0.045; APP/PS1 RNA-seq: Figure 2B, Spearman’s *R* = −0.035; 5 × FAD RNA-seq: Figure 2C, Spearman’s *R* = −0.029; 3 × Tg-AD model: Figure 2D, Spearman’s *R* = 0.012). Similar results were observed between the cortical transcriptomes of POCD and AD (5 × FAD RNA-seq: Supplementary Figure 1A, Spearman’s *R* = −0.0034; 3 × Tg-AD microarray: Supplementary Figure 1B, Spearman’s *R* = −0.038). In contrast, there was a better correlation among AD models (Supplementary Figure 2A, Spearman’s *R* = 0.17; Supplementary Figure 2B, Spearman’s *R* = 0.22). Considering that high-throughput data are often noisy, we extracted the protein-coding genes with the lowest deciles of adjusted *p*-values for subset analysis. The correlation coefficients were still consistently very low regardless of the tissue resources (hippocampus: Figures 2E–H, Spearman’s *R* = −0.13 to 0.042; cortex: Supplementary Figure 3; Spearman’s *R* = −0.057 to 0.073).

### Postoperative Cognitive Dysfunction vs. Alzheimer’s Disease: Overall Pathway-Level Comparison

**FIGURE 3:**
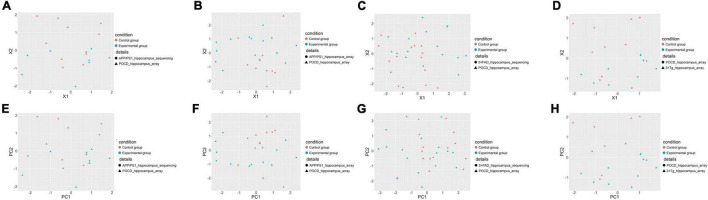
Overall pathway-level comparison of the hippocampal transcriptomic changes between POCD and different AD transgenic models visualized through principal coordinate analysis **(A–D)** and principal component analysis **(E–H)**. **(A)** GSE95426 (POCD model microarray data) vs. GSE93678 (APP/PS1 model RNA-seq data); **(B)** GSE95426 vs. GSE135999 (APP/PS1 model microarray data); **(C)** GSE95426 vs. GSE168137 (5 × FAD model RNA-seq data); **(D)** GSE95426 vs. GSE165111 (3 × Tg model microarray data). **(E)** GSE95426 (POCD model microarray data) vs. GSE93678 (APP/PS1 model RNA-seq data); **(F)** GSE95426 vs. GSE135999 (APP/PS1 model microarray data); **(G)** GSE95426 vs. GSE168137 (5 × FAD model RNA-seq data); **(H)** GSE95426 vs. GSE165111 (3 × Tg model microarray data).

The gene expression information of each sample was converted into KEGG pathway-based matrices using gene set variation analysis to facilitate an overall pathway-level comparison of the AD and POCD models. These matrices were pooled and visualized by principal coordinate analysis and principal component analysis. Theoretically, dots with the same color (the same condition), regardless of their shapes, should cluster together if the animal models have similar pathway-level changes. In contrast, there should be no clear boundary between dots of different colors if the animal models underwent divergent pathophysiological changes. As a result, the transcriptomes of POCD and AD hippocampi (Figure 3) and cortexes (Supplementary Figures 4, 5) had low pathway-level similarity. In contrast, there was higher similarity between the different AD models (Supplementary Figures 6, 7).

### Comparison of Postoperative Cognitive Dysfunction Animal Models Against the Classical Alzheimer’s Disease Signaling Pathway

**FIGURE 4:**
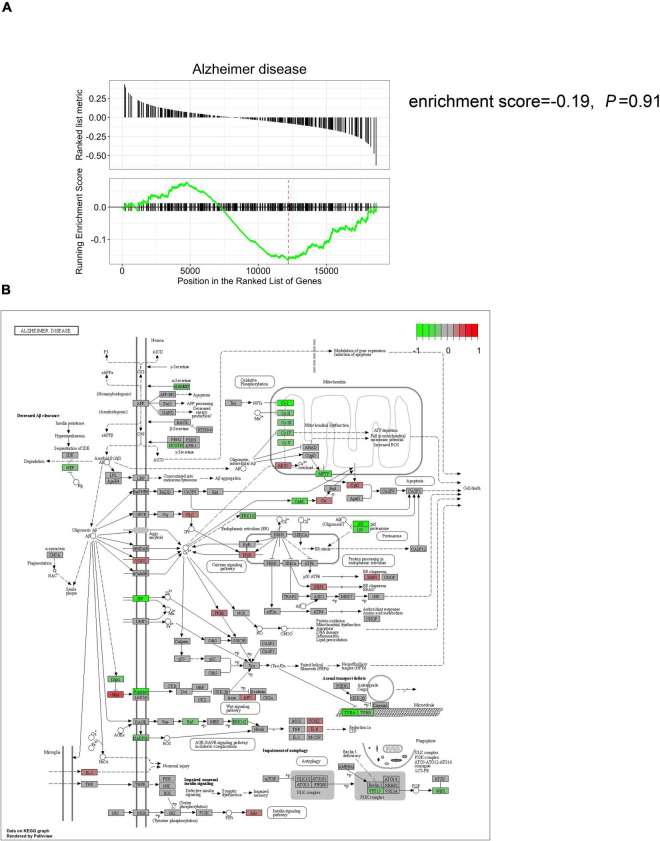
Comparison of the hippocampal gene expression pattern in POCD animal models against the classical AD signaling pathway. **(A)** Gene set enrichment analysis showed that the classical AD signaling pathway is not significantly dysregulated in the POCD hippocampus (enrichment score = –0.19, *p* = 0.91). **(B)** KEGG mapping of the dysregulated gene expression in the hippocampus (red and green: up and downregulated in POCD, respectively).

Gene set enrichment analysis was initially performed on POCD datasets using MSigDB (C2: KEGG) as the curated gene set. The classical AD signaling pathway was non-significantly dysregulated in either the POCD hippocampus or the cortex gene set (Figure 4A and Supplementary Figure 8A; HP: enrichment score = −0.19, *p* = 0.91; CX: enrichment score = −0.34, *p* = 0.6). The POCD DEG lists were mapped to the AD pathway graph using the pathview package in R. Mitochondrial complex and tubulin mRNAs were downregulated in the POCD and AD hippocampi and cortexes (Figure 4B and Supplementary Figure 8B). In addition, transcripts of some inflammatory cytokines such as interleukin-1 (IL-1) and IL-6 were upregulated in the POCD hippocampus (Figure 4B and Supplementary Figure 8B).

### Postoperative Cognitive Dysfunction vs. Alzheimer’s Disease: A Comparison of Animal Models

**FIGURE 5:**
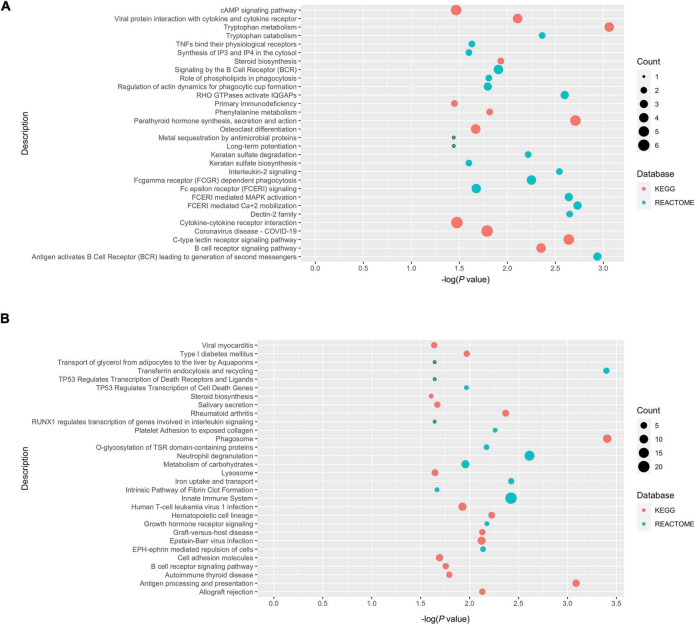
Over-presentation analysis of class 1 and class 2 genes in the POCD and AD hippocampus. **(A)** Class 1 genes (dysregulated in the same direction in POCD and AD); **(B)** class 2 genes (dysregulated in different directions).

A meta-analysis was performed using the robust rank aggregation method to obtain a more robust result of the transcriptomic changes in AD animal models, and the top 10% of the dysregulated genes ranked by *p*-values were extracted. Class 1 and class 2 genes were defined as those dysregulated in the same (Figure 5A) and different (Figure 5B) directions in POCD and AD, respectively. Over-representation analysis was then performed to explore the shared and unique molecular pathways, which were visualized using bubble plots. Class 1 genes in the hippocampus were mainly enriched in tryptophan metabolism, antigen activation of B cell receptor (BCR) leading to the generation of second messengers, and FCERI-mediated Ca^2+^ mobilization. Class 2 genes in the hippocampus were mainly enriched in the following pathways: phagosomes, transferrin endocytosis and recycling, and antigen processing and presentation. However, over-representation analysis revealed cytokine–cytokine receptor interactions and cholesterol biosynthesis were the most enriched pathway terms for class 1 and class 2 genes, respectively, in the cortex (Supplementary Figure 9). Detailed lists of enriched terms and genes are shown in the Supplementary Tables 1–4.

### Protein–Protein Interaction Network Construction and Hub Gene Identification

**FIGURE 6:**
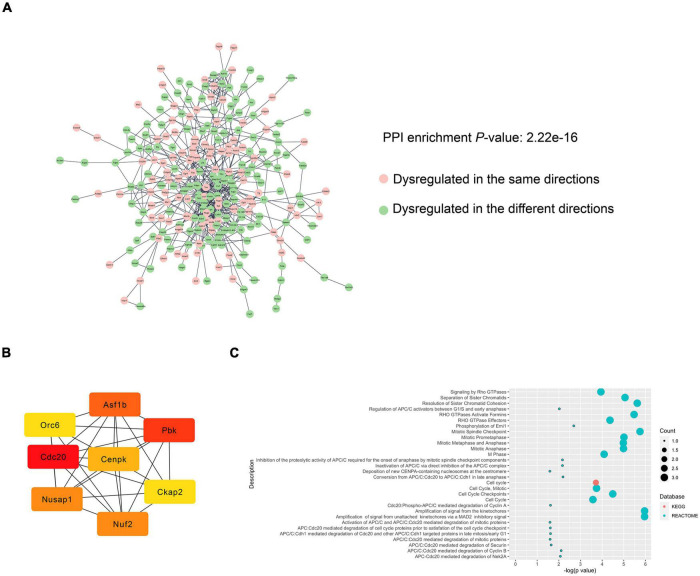
PPI analysis of the DEGs between the POCD and AD hippocampus. **(A)** PIN reconstruction of the hippocampal DEGs between POCD and AD. Red, upregulated nodes; green, downregulated nodes. **(B)** The core cluster consists of eight genes with the color shading representing the score calculated by the maximal clique centrality algorithm. **(C)** Over-presentation analysis of the core cluster.

To further investigate the relationship between POCD and AD in terms of the dysregulated genes, PINs were constructed based on the String database. Only edges with a combined score > 0.4 were included in the topological analysis. Networks of the hippocampus (Figure 6A) and cortex (Supplementary Figure 10) had significantly more interactions than expected, indicating that the genes were biologically connected (average local clustering coefficient for the network of hippocampus: 0.343; PPI enrichment value for the hippocampal network: 2.22e^–16^; average local clustering coefficient for the cortical network: 0.357, PPI enrichment value for the cortical network: 4.44e^–15^). The cluster comprising the genes with the highest scores calculated by the maximal clique centrality algorithm was recognized as the core cluster for each network (Figure 6B). Over-representation analysis showed that the core hippocampus cluster was mainly enriched in mitosis-related pathways (Figure 6C). The hub genes in the cortex network were mainly associated with cholesterol biosynthesis and steroid metabolism (Supplementary Figure 11).

### Isoflurane Anesthesia and Exploratory Laparotomy Induced Postoperative Cognitive Dysfunction in Aged Mice

**FIGURE 7:**
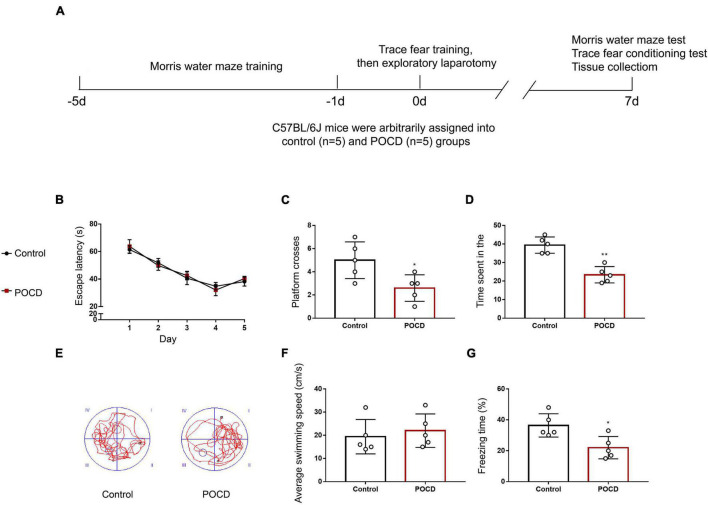
Exploratory laparotomy successfully induces POCD in aged mice. **(A)** Diagram showing the timeline for behavioral tests and the operation. **(B)** Average escape latency for the training phase in the Morris water maze (MWM) test. **(C)** The number of platform-crossing events in the probe trial of the MWM. **(D)** The time spent in the target quadrant in the probe trial of the MWM. **(E)** Representative swimming traces of mice during the probe trial in the MWM test. **(F)** Swimming speed during the probe trial of the MWM. **(G)** Freezing time in the trace fear conditioning (TFC) test. All experiments were repeated three times. *p < 0.05, **p < 0.01 vs. control group. The data are presented as the mean ± SEM (*n* = 5).

A diagram showing the timeline for behavioral tests is briefly presented in Figure 7A. There were daily improvements in the MWM latency during the early training phase, then a plateau was reached. No significant difference was found in the escape latency between the control and POCD group (Figure 7B). During the probe test, the number of platform-crossing events and the time spent in the target quadrant were remarkably reduced in the POCD group compared with those in the control group (Figures 7C–E). No significant difference in swimming speed was observed between control and POCD groups (Figure 7F). In the TFC test, mice in the POCD group exhibited less freezing behavior than those in the control group (Figure 7G). Taken together, isoflurane anesthesia and exploratory laparotomy successfully induced POCD in aged mice.

### Verification of Hippocampal mRNA Expression via Quantitative PCR

**FIGURE 8:**
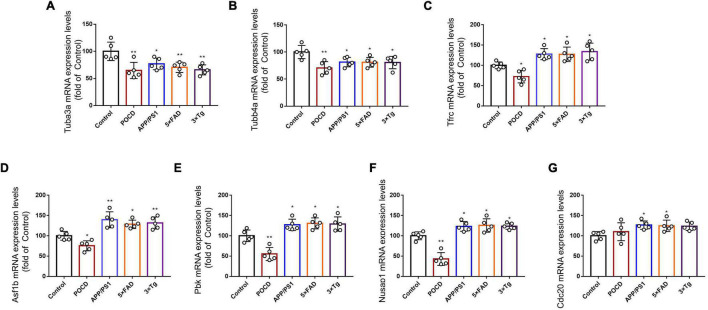
Expression levels of **(A)** *Tuba3a*, **(B)** *Tubb4a*, **(C)** *Tfrc*, **(D)** *Asf1*, **(E)** *Pbk*, **(F)** *Nusap1*, and **(G)** *Cdc20* mRNA detected by qPCR in control, POCD, and AD groups. All experiments were repeated three times. *p < 0.05, **p < 0.01 vs. control group. The data are presented as the mean ± SEM (*n* = 5).

The expression of 18 hippocampal mRNAs of interest was measured using qPCR in control, POCD, and AD mice. Tubulin-related mRNAs (*Tuba3a* and *Tubb4a*) were significantly downregulated in both the POCD and AD hippocampi (Figures 8A,B). *Tfrc* mRNA, which encodes the transferrin receptor, responsible for cellular iron uptake, was upregulated in the AD but downregulated in the POCD hippocampus (Figure 8C). This phenomenon was also observed in terms of mitosis-related mRNAs including *Asf1b*, *Pbk*, and *Nusap1* (Figures 8D–F). Several genes encoding mitochondrial complex I or II (*Ndufs1*, *Sdhb*, *Sdhc*) were downregulated in the POCD hippocampus (Supplementary Figures 12A–F). However, we found that *Cdc20* mRNA, another mitosis-related mRNA, was only slightly upregulated in the POCD hippocampus with no statistical significance (Figure 8G). Both POCD and AD increased the expression of inflammatory cytokine mRNAs (*Il1* and *Il6*), as well as tryptophan metabolism-related mRNAs (*Haao* and *Lao1*) (Supplementary Figures 12G–J).

## Discussion

Postoperative cognitive dysfunction and AD are both characterized by cognitive dysfunction and have a high prevalence among the elderly (Lin et al., 2020). It is reasonable to postulate that these two diseases might share some common pathological pathways (Fodale et al., 2010). Previous studies have hypothesized that the aberrant function of the cholinergic system and Aβ accumulation are important in AD and POCD (Xie and Tanzi, 2006). However, clinical trials have not observed significant cognition deterioration in patients with AD after surgery or anesthesia, indicating that AD and POCD are quite different in some respects (Seitz et al., 2011; Liu et al., 2013; Steinmetz et al., 2013). Therefore, the aim of this study was to determine the transcriptomic similarity between POCD and AD. To our knowledge, this is the first systematic study on this topic.

The macro-level comparison of all protein-coding transcriptomic changes between POCD and AD revealed a very weak correlation. Further correlation analysis using the dysregulated transcripts with the lowest *p*-values showed a similar result. Pathway-level analysis using gene set variation analysis showed that POCD and AD animal models are different from a biological perspective. Finally, gene set enrichment analysis showed that the classical AD signaling pathway was not significantly dysregulated in POCD gene sets. Overall, these data support the notion that AD and POCD are two disease entities despite their similar clinical manifestations.

Then, we compared the transcriptome of POCD against the classical AD signaling pathway, which revealed that mRNA levels encoding the inflammatory cytokines IL-1 and IL-6 were upregulated in the POCD and AD hippocampus. This is consistent with the well-accepted theory that neuroinflammation is a driving force in POCD development, especially in the hippocampus (Alam et al., 2018; Subramaniyan and Terrando, 2019). Moreover, hippocampal mRNAs of mitochondrial respiratory chain complexes were downregulated in POCD, which is consistent with results of previous studies (Netto et al., 2018; Wei et al., 2019). A reduction in mitochondrial complexes I, II, II, IV, and V has been intensively reported in human AD (Fisar et al., 2019). Mitochondrial respiratory chain deficiencies can cause neuronal degeneration and cell death resulting from oxidative damage and energy detection. Taken together, mitochondrial respiratory chain dysfunction might be a noticeable feature of POCD, and targeted therapies using PPAR-c and PGC-1a agonists previously developed for AD treatment might yield a protective effect in POCD (Kim et al., 2007; Golpich et al., 2017). Another finding of our study is that the expression of microtubule-related mRNAs (*Tuba* and *Tubb*) is decreased in the POCD and AD hippocampi. Microtubule loss, reduced tubulin acetylation, and subsequent axonal transport defects occur during the early preclinical stages of AD (Vicario-Orri et al., 2015). There is increasing evidence of several anesthetics exerting their effects via direct binding to tubulin (Craddock et al., 2017). Furthermore, proteomic analysis showed that tubulin gene expression was prominently altered following exposure to volatile anesthetics (Pan et al., 2008). Therefore, the reduced level of tubulin and concomitant microtubule disassembly might be a common therapeutic target for AD and POCD. In this regard, paclitaxel and its analogs, which show efficacy in preventing Aβ accumulation in AD models, are promising to have a protective effect on POCD (Michaelis et al., 2005; Rice et al., 2005; Trojanowski et al., 2005).

Next, we made a direct comparison of the animal models. As a result, POCD and AD might share similar pathways, such as tryptophan metabolism. Tryptophan metabolism plays a pivotal role in the synthesis of 5-hydroxytryptamine (serotonin), which modulates a wide array of cognitive processes (Silber and Schmitt, 2010). Emerging evidence shows that increased tryptophan plasma levels improve learning and memory in patients with AD, along with a concomitant change in 5-hydroxytryptamine synthesis (Maitre et al., 2020). However, there is a lack of research on the relationship between POCD and tryptophan metabolism, which deserves future investigation. Nevertheless, POCD and AD might be associated with different pathological changes in transferrin-related pathways. We observed increased expression of *Tfrc* in the hippocampus of AD animal models, which is associated with excessive iron accumulation and Aβ deposition (Johnstone et al., 2012; Banerjee et al., 2016). However, *Tfrc* was downregulated in POCD indicating decreased iron uptake. Considering the important role of iron metabolism in cognitive function, its pathological significance warrants further investigation.

We also performed PPI analysis to reveal the intrinsic relationship between the DEGs of AD and POCD. PPI analysis showed that the dysregulated genes in AD and POCD formed biologically connected networks in the hippocampus and cortex. Enrichment analysis of the core clusters highlighted the central role of mitosis-related genes in the hippocampus. It is suggested that mitotic dysfunction is implicated in AD onset and contributes to neurodegeneration (van Leeuwen and Hoozemans, 2015; Varghese et al., 2016). Consistent with previous reports, most genes in the core cluster were upregulated in the hippocampus of patients with AD. Only one of these eight genes, namely *Cdc20*, was slightly upregulated in the POCD hippocampal region. Further research is required to determine whether this phenomenon indicates a potential role for mitosis errors in POCD development.

Despite these findings, our analysis was based on transgenic animal models of AD. These mouse models can recapitulate familial early-onset AD (EOAD) (Bettens et al., 2013). However, sporadic late-onset AD (LOAD) accounts for the majority of AD cases (Sasaguri et al., 2017). Because EOAD and LOAD phenocopy each other clinically and histologically, the amyloid hypothesis—although based on molecular defects isolated in EOAD—was plausibly proposed to underlie all forms of AD (Small and Duff, 2008). Although inconsistencies exist between sporadic and familial AD, clinical findings from a growing number of Aβ-reducing drug trials in LOAD suggest that transgenic models linking Aβ with tau are worth considering and biologically plausible (Geschwind, 2003). Furthermore, Forner et al. (2021) found that the gene expression profile of 18 months 5 × FAD mice can better recapitulate the human AD brain than those with younger age (Forner et al., 2021), supporting the use of aged transgenic animal models. However, future studies are still required to gain a better knowledge of the extent to which these models actually reproduce sporadic LOAD.

In addition to the above points, our study has some technical limitations. First, we focused on only transcriptomic data; therefore, future studies should include proteomic and metabolomic data to provide a more comprehensive understanding of the molecular mechanisms involved. Second, a limited amount of POCD high-throughput data was identified compared with that for AD, which might have introduced a bias into our results. Third, mRNA expression, rather than protein expression, was used to reconstruct the protein–protein interaction network, which might not reflect the actual situation. In conclusion, our study revealed the unique and shared molecular mechanisms between POCD and AD and provides a valuable resource for biomarker and therapeutic target discovery. Some of our findings provide viable and promising treatment targets for POCD and provide a strategy for investments in long-term, well-planned, early intervention trials for POCD.

## Data Availability Statement

The datasets presented in this study can be found in online repositories. The names of the repository/repositories and accession number(s) can be found in this article/Supplementary Material.

## Ethics Statement

The animal study was reviewed and approved by the Institutional Animal Care and Use Committee (Approval No.: 2003021) and the Laboratory Animal Ethics Committee of Nanjing Medical University.

## Author Contributions

L-TZ and XZ designed the study, wrote the protocol, and were the guarantors of this work and had full access to the data. LW managed the literature searches and analyses. Y-WW undertook the statistical analysis and wrote the first draft of the manuscript. S-JY and YZ performed the animal experiments. All authors contributed to the article and approved the submitted version.

## Conflict of Interest

The authors declare that the research was conducted in the absence of any commercial or financial relationships that could be construed as a potential conflict of interest.

## Publisher’s Note

All claims expressed in this article are solely those of the authors and do not necessarily represent those of their affiliated organizations, or those of the publisher, the editors and the reviewers. Any product that may be evaluated in this article, or claim that may be made by its manufacturer, is not guaranteed or endorsed by the publisher.

## Footnotes

1
https://www.ncbi.nlm.nih.gov/geo/

2
https://www.ebi.ac.uk/arrayexpress/

3
https://db.cngb.org/

4
https://www.ddbj.nig.ac.jp/index-e.html

5
http://geneontology.org/

6
http://www.genome.jp/kegg

7
http://www.reactome.org

8
https://string-db.org/
