# Supplementary material for: Postoperative Cognitive Dysfunction and Alzheimer’s Disease: A Transcriptome-Based Comparison of Animal Models
Source: Front Aging Neurosci. 2022 Jun 28;14:900350. doi: 10.3389/fnagi.2022.900350 (PMC9273890; doi:10.3389/fnagi.2022.900350)
Supplement: Supplementary file 5 [file Data_Sheet_1.PDF]

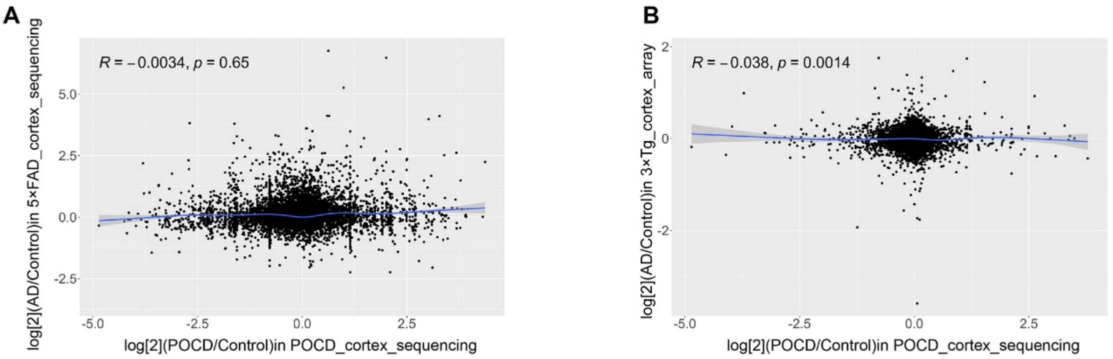

Supplementary Figure 1. Correlation analysis between POCD and different AD models of all cortical protein-coding gene expression. (A) GSE174412 (POCD model RNA-seq data) vs. GSE168137 (5×FAD model RNA-seq data); (B) GSE174412 vs. GSE60911 (3×Tg model microarray data).

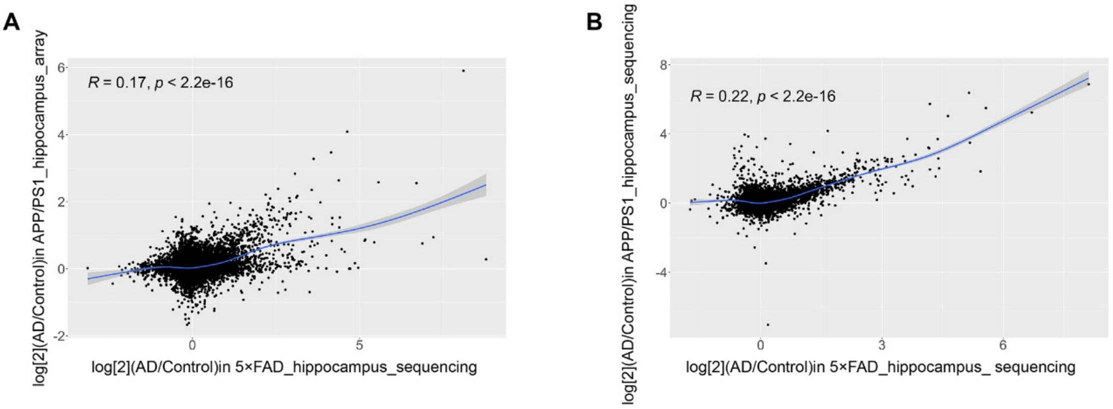

Supplementary Figure 2. Correlation analysis of all hippocampal protein-coding gene expression among different AD models. (A) GSE168137 (5×FAD model RNA-seq data) vs. GSE135999 (APP/PS1 model microarray data); (B) GSE168137 (5×FAD model RNA-seq data) vs. GSE93678 (APP/PS1 model RNA-seq data).

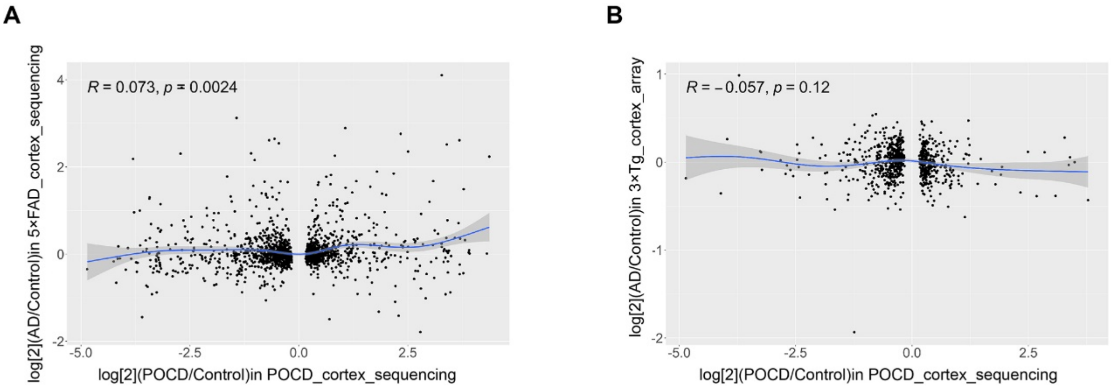

Supplementary Figure 3. Correlation analysis of significantly dysregulated gene expression between POCD and different AD models in the cortex. (A) GSE174412 (POCD model RNA-seq data) vs. GSE168137 (5×FAD model RNA-seq data); (B) GSE174412 (POCD model RNA-seq data) vs. GSE60911(3×Tg model microarray data).

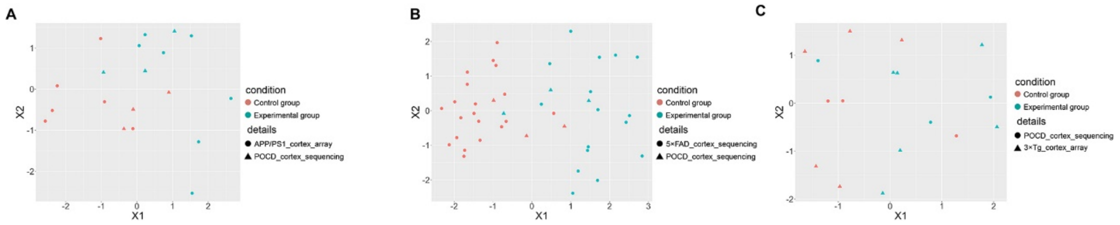

Supplementary Figure 4. Overall pathway-level comparison of cortical transcriptomic changes between POCD and different AD models visualized by principal coordinate analysis. (A) GSE174412 (POCD model RNA-seq data) vs. GSE135999 (APP/PS1 model microarray data); (B) GSE174412 vs. GSE168137 (5×FAD model RNA-seq data); (C) GSE174412 vs. GSE60911 (3×Tg model microarray data).

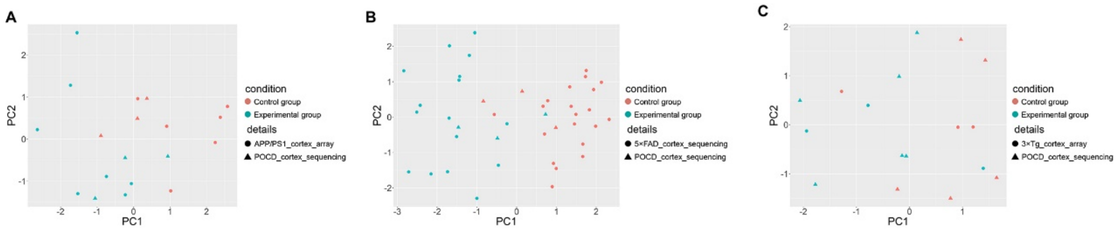

Supplementary Figure 5. Overall pathway-level comparison of cortical transcriptomic changes between POCD and different AD models visualized by principal component analyses. (A) GSE174412 (POCD model RNA-seq data) vs. GSE135999 (APP/PS1 model microarray data); (B) GSE174412 vs. GSE168137 (5×FAD model RNA-seq data); (C) GSE174412 vs. GSE60911 (3×Tg model microarray data).

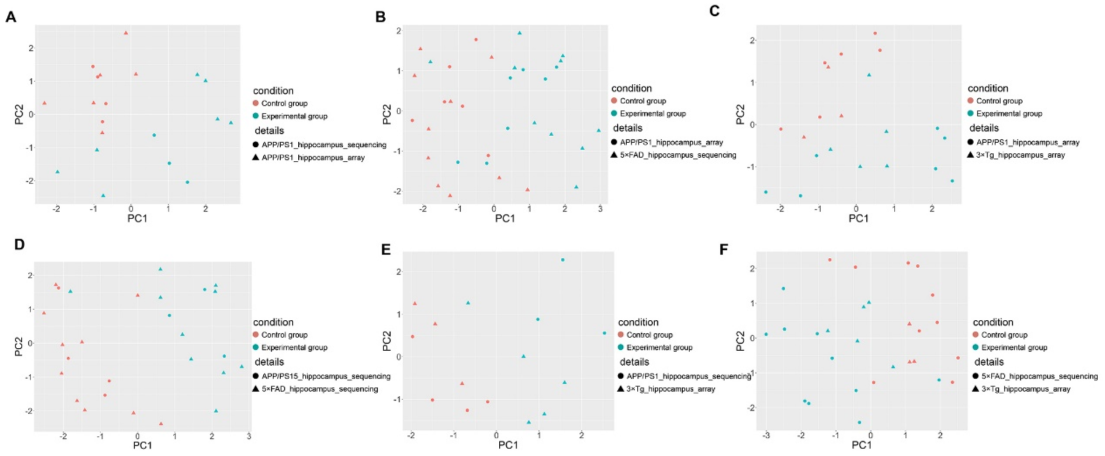

Supplementary Figure 6. Overall pathway-level comparison of hippocampal transcriptomic changes within different AD models visualized by principal component analyses. (A) GSE135999 (APP/PS1 model microarray data) vs. GSE93678 (APP/PS1 model RNA-seq data); (B) GSE135999 vs. GSE168137 (5×FAD model RNA-seq data); (C) GSE135999 vs. GSE165111 (3×Tg model microarray data); (D) GSE93678 vs. GSE168137; E: GSE93678 vs. GSE165111; F: GSE168137 vs. GSE165111.

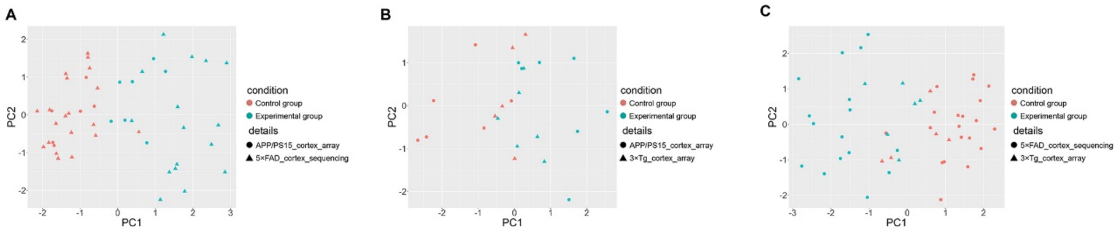

Supplementary Figure 7. Overall pathway-level comparison of the cortical transcriptomic changes within different AD models visualized by principal component analyses. (A) GSE135999 (APP/PS1 model microarray data) vs. GSE168137 (5×FAD model RNA-seq data); (B) GSE135999 vs. GSE60911 (3×Tg model microarray data); (C) GSE168137 vs. GSE60911.

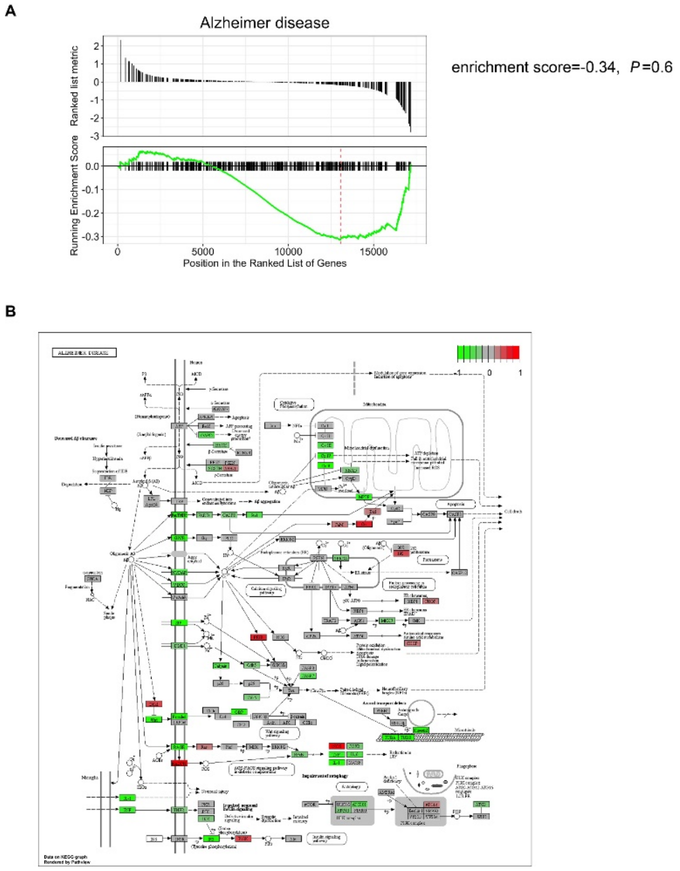

Supplementary Figure 8. Comparison of the cortical gene expression pattern in POCD animal models against the classical AD signaling pathway. (A) Gene set enrichment analysis showed that the classical AD signaling pathway is not significantly dysregulated in the POCD cortex (enrichment score = –0.34, *P* = 0.6). (B) KEGG mapping of the dysregulated gene expressions in the cortex (red and green: upregulated and downregulated genes of POCD compared with the classical AD signaling pathway, respectively).

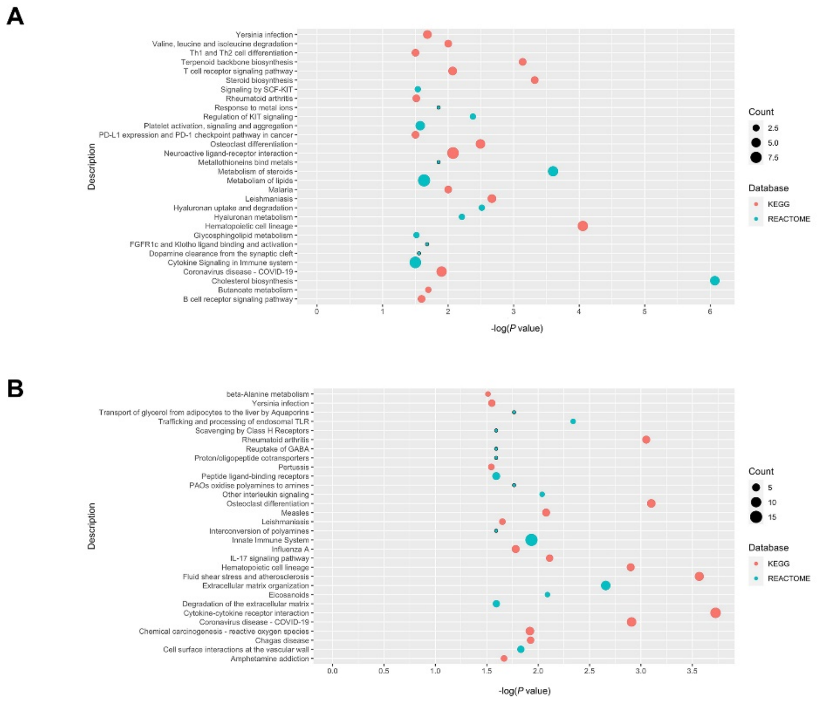

Supplementary Figure 9. Over-presentation analysis of class 1 and class 2 genes in the POCD and AD cortex. (A) Class 1 genes (dysregulated in the same direction in POCD and AD); (B) class 2 genes (dysregulated in different directions).

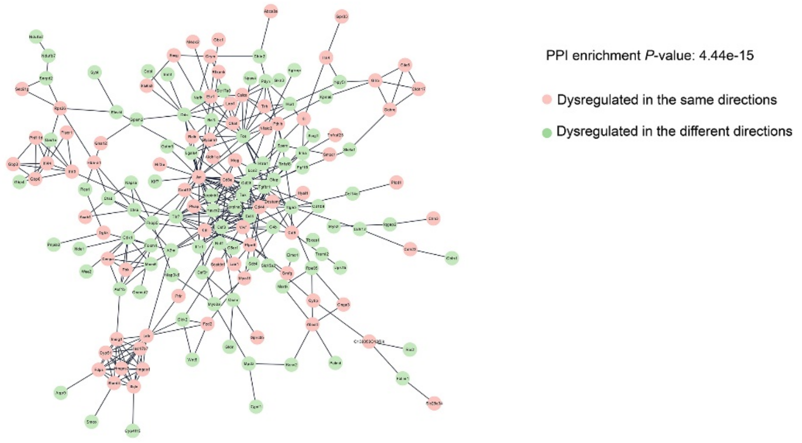

Supplementary Figure 10. Protein–protein interaction network reconstruction of dysregulated genes in the POCD and AD cortex.

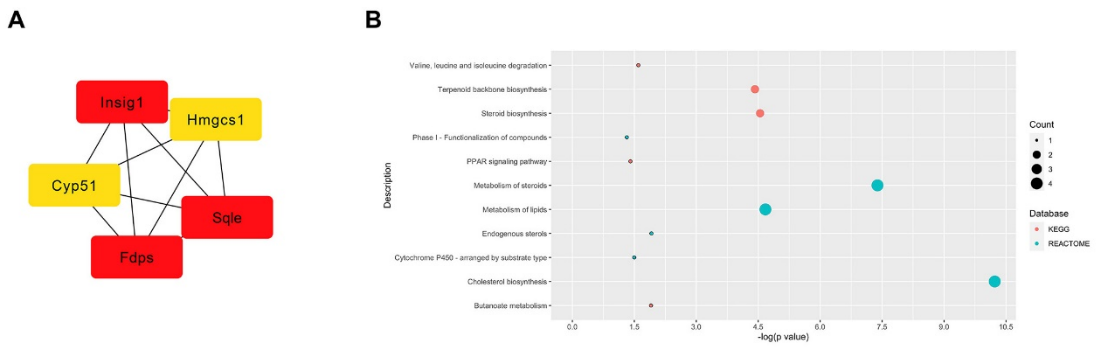

Supplementary Figure 11. Core cluster identification from the protein–protein interaction network in the cortex. (A)The core cluster is consisted of 8 genes with the shade of the color representing the score calculated by the maximal clique centrality algorithm. (B) Over-presentation analysis of the core cluster.

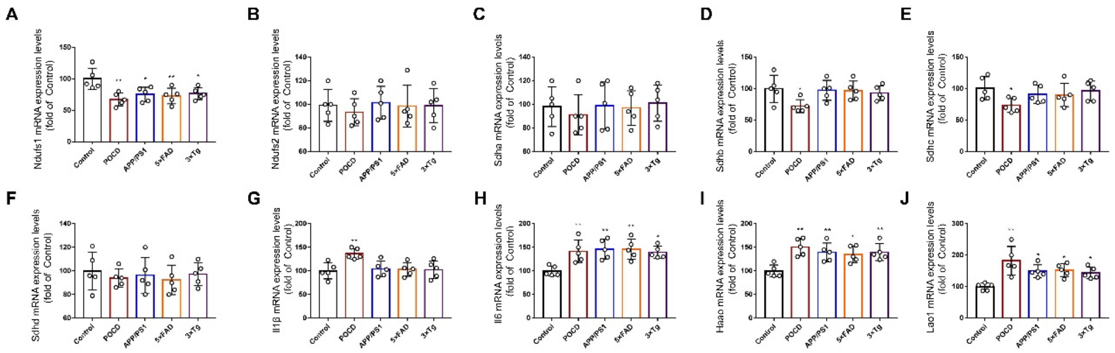

Supplementary Figure 12. The mRNA expression levels of (A) *Ndufs1*, (B) *Ndufs2*, (C) *Sdha*, (D) *Sdhb*, (E) *Sdhc*, (F) *Sdhd*, (G) *Il1β*, (H) *Il6*, (I) *Haao* and (J) *Lao1* mRNA detected by qPCR in control, POCD and AD groups. $^{*}P$<0.05, $^{**}P$ < 0.01 vs. control group. The data are presented as the mean ± SEM (n=5).
