# Supplementary material for: Postoperative Cognitive Dysfunction and Alzheimer’s Disease: A Transcriptome-Based Comparison of Animal Models
Source: Front Aging Neurosci. 2022 Jun 28;14:900350. doi: 10.3389/fnagi.2022.900350 (PMC9273890; doi:10.3389/fnagi.2022.900350)
Supplement: Supplementary file 6 [file Data_Sheet_2.PDF]

Animals and Surgery

Male C57BL/6j mice (RRID: IMSR_JAX:000664) and congenic 5×FAD mice (RRID: MMRRC_034848-MU), 3×TgAD mice (RRID: MMRRC_034830-MU), APP/PS1 mice (RRID: MMRRC_034832-MU) were provided by the Jiangsu Animal Experimental for Medical and Pharmaceutical Research Center. All animals were housed in standard mouse cages (27.94 × 15.24 × 11.43 cm) with 3-4 individuals in each cage and with *ad libitum* access to food and water until 12 months of age. The cages were kept in a quiet, 12-hour light/dark cycle standard. After acclimation for at least 1 week, animals were used in experiments. All animal experiments in this study were approved by the Institutional Animal Care and Use Committee (Approval No.: 2003021) and the Laboratory Animal Ethics Committee of Nanjing Medical University.

The total number of mice used for experimental analysis was 25, including C57BL/6j (n=10, male,12-month-old, mean weight: 30.4 g), congenic 5×FAD (n=5, male,12-month-old, mean weight: 25.2 g), 3×TgAD (n=5, male,12-month-old, mean weight: 23.7g), and APP/PS1 (n=5, male,12-month-old, mean weight: 26.9 g) mice. No exclusion criteria were predetermined and no animals were excluded. No animals died during the experiments.

Exploratory laparotomy under isoflurane anesthesia was used to construct the POCD model (Qiu *et al.* 2020). Male C57BL/6j mice (12-months-old) were arbitrarily assigned into control (n=5) and POCD (n=5) groups (simple randomization). Briefly, after arrival animals were arbitrarily allocated to numbered cages by an independent technician, who was not involved in the experiments. The random allocation sequence to experimental groups was generated using chit method. A complete pool of chits, each bearing a group description (e.g. "POCD"), was prepared, mixed and then drawn from a box, creating a random sequence that was next matched with numbered animals. Male C57BL/6j mice from POCD group were anesthetized under isoflurane anesthesia (1.5 % isoflurane mixed with oxygen at 2 L/minute) for 30 minutes before exploratory laparotomy. Isoflurane (Cat#−792632-Sigma-Aldrich, Saint Louis, MO-2020) was used as the anesthesia of choice for its rapid onset and quick recovery; thereby minimizing pain. The degree of anesthesia was verified by the absence of pinch reflex. Under full aseptic conditions, a median abdominal incision approximately 1-2 cm was made to enter the abdominal cavity and explore the abdominal organs such as the intestine, musculature, and viscera. The abdominal wound was irrigated and the skin was sutured with Sterile 5-0. In order to minimize the suffering of animals, qualified and experienced laboratory personnel will handle these animals very carefully, and at the end of the procedure and the following three days, 2.5% lidocaine cream was applied to the incision to alleviate surgery-associated pain. Male C57BL/6j mice from control group, 5×FAD mice, APP/PS1 mice and 3×Tg AD mice did not receive any prior treatment and surgery. The study was not pre-registered. Our experiment conformed with the ARRIVE guidelines regarding the care and use of animals for experimental procedures. This study was exploratory, and there was no pre-specified endpoint. No method of calculation to predetermine sample size was employed in the study.

Behavioral tests

Morris water maze (MWM) test

MWM was used to assess spatial working memory as previously described (Mifflin et al., 2021). The MWM was conducted in a round white pool 94 cm in diameter and 31 cm deep. The pool in the Morris Water Labyrinth (Shanghai Xinsoft Information Technology Co., Ltd., China) was

filled to a depth of 30 cm with water made opaque with nontoxic white paint to hide the platform. The platform (25 cm^2 plexiglas square, 15 cm in diameter) was placed in the center of one quadrant of the pool, and submerged 1cm beneath the surface of the water, which was maintained at 25 ± 0.5°C throughout testing. C57BL/6j mice were subjected to training trials for 5 consecutive days before exploratory laparotomy. The platform remained in the same quadrant for all mice throughout the learning trials, but the start location was pseudo-randomly selected. A trial began by placing the mouse on the platform for 20 seconds to encode extra-maze cues. Each mouse was given 60 seconds to locate the hidden platform. If the animal failed to reach the hidden platform in the 60 seconds, they were gently guided to the location and allowed to re-orient to the distal visual cues for an additional 20 seconds before being removed from the pool. After removal from the pool, mice were manually dried with a terrycloth towel and placed in a warming cage for at least 30 minutes to ensure thorough dryness. A probe trial was conducted one-week post exploratory laparotomy. The mice were allowed to swim freely for 60 seconds before the probe test. During the probe test, the platform was removed so that the mice could locate the platform using a local visual stimulus rather than relying on spatial orientation to extra-maze cues. The mice were allowed to swim freely for 60 seconds. All trials were video recorded, and the data was analyzed via the SMART digital tracking system (Version 2.5, Panlab, Barcelona, Spain). The dependent variables used for the analysis were latency (seconds) to the platform for the learning trials and number of platform crossings, latency to first cross the platform location (seconds), and velocity (cm/second) for the probe trial.

Trace fear conditioning (TFC) test

Hippocampus-dependent memory was evaluated by TFC test as previously described (Wang et al., 2017). Complete methodological details are described as follow according to our previous study. C57BL/6j mice from POCD and control group were trained to associate an environment (context) with tone and foot shock 4-5 hours before exploratory laparotomy. The training consisted of placing the mice in the conditioning chamber and allowing exploration of the surroundings for 180 seconds. Next, an auditory cue (70 dB) was presented for 30 seconds and a foot shock (0.7 mA) was administered after termination of the tone. This procedure was repeated with an interval of 60 seconds, and the mice were removed from the chamber after 3 cycles. Contextual assessment was performed at 7 days post-operation in the same chamber but with no cues (tone or shock). Freezing behavior, recognized as lack of movement, was recorded for 360 seconds by video and analyzed using software (Xeye Fcs, Beijing MacroAmbition S&T Development Co., Ltd., Beijing, China). A decrease in the percentage of time spent frozen indicated impairment of hippocampus-independent memory. During each test interval, the conditioning chamber was cleaned with 75% ethanol to eliminate feces and odors.

Tissue processing

One hour after the behavioral tests at 7 days after exploratory laparotomy, all animals (C57BL/6j, 5×FAD, APP/PS1 and 3×Tg mice) were anesthetized with 1% sodium pentobarbital (Cat#11715-Sigma-Aldrich, Saint Louis, MO-2020) by intraperitoneal injection for 30 minutes. When squeezing the hind toes several times using tweezers, no action response suggests that the mouse has lost consciousness. Then, mice were quickly euthanized by exsanguination. The brain was

extracted to obtain the hippocampus; the tissues were then frozen in liquid nitrogen for RNA isolation and reverse transcription.

Quantitative PCR

Total RNA from the hippocampus was extracted with TRIzol reagent (Cat#15596026-Invitrogen, Carlsbad, CA-2020), and reverse transcription was performed using the SuperScript III first strand synthesis system (Invitrogen, Carlsbad, CA). The concentration and purity of the resultant cDNA were determined using a Nanodrop spectrophotometer (NanoDrop, Wilmington, DE). Quantitative PCR (qPCR) amplification was performed using the STEP ONE Real Time PCR Detection System (Applied Biosystems, Foster City, CA) with SYBR Green master mix (Cat#43-091-55-Applied Biosystems, Foster City, CA-2021) at a final volume of 10 μl that contained 1 μl cDNA template from each sample. The PCR was carried out using the following cycling protocol: a 95 °C denaturation step for 5 minutes followed by 40 cycles of 95 °C denaturation (15 seconds), 60 °C annealing (15 seconds), and 60 °C extension (30 seconds). Detection of the fluorescent product was carried out at the end of each 60 °C extension period. The relative mRNA values were normalized to the Gapdh gene control values and calculated using the comparative cycle threshold (ΔΔCt) method.

Primers are listed as the following:

mouse Tuba3a-forward, 5′-GTCCTGGATCGAATCCGAAAG-3′,
Tuba3a-reverse, 5′-CGGAATGCTCTAGGGTGGTG-3′,
mouse Tubb4a- forward, 5′-CACCTGCCTACGTTTCCCG-3′,
Tubb4a-reverse, 5′-GGTGCGAATCCTGGCATGA-3′,
mouse Tfrc-forward, 5′-GTTTCTGCCAGCCCCTTATTAT-3′,
Tfrc-reverse, 5′-GCAAGGAAAGGATATGCAGCA-3′,
mouse Asf1b-forward, 5′- CCCTTCCGGTTCGAGATCAG-3′,
Asf1b-reverse, 5′-GGCGTCCGTCTCAGGAATG-3′,
mouse Pbk-forward, 5′-TGGGCCGTGAAAAAGATAAGTC-3′,
Pbk-reverse, 5′-CTGGCTTCAGTAAAAGCACGATA-3′,
mouse Nusap1-forward, 5′-TAAGTACAGCGACCTGCAAAAT-3′,
Nusap1-reverse, 5′- CTCACTGCTAACGTGTATCTCAG-3′,
mouse Cdc20-forward, 5′-TTCGTGTTCGAGAGCGATTTG-3′,
Cdc20-reverse, 5′-ACCTTGGAACTAGATTTGCCAG-3′,
mouse Gapdh-forward, 5′-AGGTCGGTGTGAACGGATTTG-3′,
Gapdh-reverse, 5′-GGGGTCGTTGATGGCAACA-3′,
mouse Ndufs1-forward, 5′-TAGCAAATCACCCATTGGATTGT-3′,
Ndufs1-reverse, 5′-CACCGGGTACACTGGATGC-3′,
mouse Ndufs2- forward, 5′-TTTCGGGAGCTGTCATGTACC-3′,
Ndufs2-reverse, 5′-TGGTCACCGCTTTTTCCTTCA-3′,
mouse Sdha-forward, 5′-GAACACTCCAAAAACAGACCTGC-3′,
Sdha-reverse, 5′-TCCACCACTGGGTATTGAGTAG-3′,
mouse Sdhb-forward, 5′-ATTTACCGATGGGACCCAGAC-3′,
Sdhb-reverse, 5′-GTCCGCACTTATTCAGATCCAC-3′,
mouse Sdhc-forward, 5′-CCCACCTGAATGCTCAGCTTT-3′,
Sdhc-reverse, 5′- AGAGGACGGTTTGAACTCGTG-3′,

mouse Sdhd-forward, 5´- TGGTCAGACCCGCTTATGTG-3´,
Sdhd-reverse, 5´- GAGCAGGGATTCAAGTACCCA-3´,
mouse Il1β-forward, 5´-GAAATGCCACCTTTTGACAGTG -3´,
Il1β-reverse, 5´-TGGATGCTCTCATCAGGACAG -3´,
mouse Il6-forward, 5´-CTGCAAGAGACTTCCATCCAG-3´,
Il6-reverse, 5´-AGTGGTATAGACAGGTCTGTTGG-3´,
mouse Haao-forward, 5´-GAACGCCGTGTGAGAGTGAA-3´,
Haao-reverse, 5´-CTCCAACGAACATGATTTTGAGC-3´,
mouse Lao1-forward, 5´-CCAGAATGGGCTCCATACCTC-3´,
Lao1-reverse, 5´-TCCAAGATGGTTACCTCGTGA-3´.

References

Mifflin, M.A., Winslow, W., Surendra, L., Tallino, S., Vural, A., and Velazquez, R. (2021). Sex differences in the IntelliCage and the Morris water maze in the APP/PS1 mouse model of amyloidosis. *Neurobiol Aging* 101**,** 130-140. doi: 10.1016/j.neurobiolaging.2021.01.018.

Wang, Y.W., Zhou, Q., Zhang, X., Qian, Q.Q., Xu, J.W., Ni, P.F., et al. (2017). Mild endoplasmic reticulum stress ameliorates lipopolysaccharide-induced neuroinflammation and cognitive impairment via regulation of microglial polarization. *J Neuroinflammation* 14(1)**,** 233. doi: 10.1186/s12974-017-1002-7.
